# Contact ability based topology control for predictable delay-tolerant networks

**DOI:** 10.1038/s41598-021-01864-5

**Published:** 2021-11-19

**Authors:** Hongsheng Chen, Chunhui Wu

**Affiliations:** School of Computer Science and Technology, Hubei University of Science and Technology, Xianning, 437100 China

**Keywords:** Energy science and technology, Engineering

## Abstract

In predictable delay tolerant networks (PDTNs), the network topology is known a priori or can be predicted over time, such as space planet networks and vehicular networks based on public buses or trains. Due to the intermittent connectivity, network partitioning, and long delays in PDTNs, most of the researchers mainly focuses on routing and data access research. However, topology control can improve energy effectiveness and increase the communication capacity, thus how to maintain the dynamic topology of PDTNs becomes crucial. In this paper, a contact ability based topology control method for PDTNs is proposed. First, the contact ability is calculated using our contact ability calculation model, and then the PDTNs is modeled as an undirected weighted contact graph which includes spatial and contact ability information. The topology control problem is defined as constructing a minimum spanning tree (MST) that the contact ability of the MST is maximized. We propose two algorithms based on undirected weighted contact graph to solve the defined problem, and compare them with the latest method in terms of energy cost and contact ability. Extensive simulation experiments demonstrate that the proposed algorithms can guarantee data transmission effectively, and reduce the network energy consumption significantly.

## Introduction

In recent years, Predictable Delay/Disruption Tolerant Networks (PDTNs) have attracted more and more researchers’ attention. It is a kind of DTNs^1,2^ whose topology is known a priori or can be predicted over time, such as vehicular networks based on public buses or taxi cabs, pocket switched networks based on human mobility and space satellite networks.

Due to the intermittent connectivity, network partitioning, long delays and node mobility, the PDTNs are not always connected, and the data is delivered in a long delay. The topology dynamically changes over time. Therefore, how to transmit data in each pair nodes successfully is very important. Many routing protocols for PDTNs transmit data from the source to the destination by utilizing opportunistically existing and time varying routing paths. However, the routing protocols only ensure transmit data from the source to the destination using some kinds of redundancy and exploit routing opportunity greedily, which may lead to unnecessary resource consumption.

Generally, topology control can avoid extra resource consumption in data transmitting, which can maintain network connectivity while minimizing the energy consumption and reducing radio interference. It is also true in DTNs, especially for PDTNs^3^. In PDTNs, a cyclic time Ct exists, and in each Ct, the network topology changes are identical. Pocket switched networks based on human mobility^4,5^, vehicular networks based on public buses or taxi cabs^6,7^, disaster-relief networks, and space communication networks^8–10^ are all PDTNs. Thus, it is not necessary to use all possible transmitting opportunities, we can use topology control mechanism to select certain transmitting opportunities to delivery data successfully in such network with lower resource consumption, especially energy consumption and connectivity.

However, it is not feasible to apply the existing topology control mechanism directly to PDTNs because the topology of PDTNs changes dynamically over time. As far as we know, Huang et al.^11–14^ and Chen et al.^15,16^ studied topology control in PDTNs. Yan et al.^17,18^ and Zhang et al.^19^ are also studied topology control in PDTNs. Taking into account temporal characteristics, they modeled PDTNs as a directed space–time graph containing spatial and temporal information. In our previously research of the literature^15^, we also considered the probabilistic connections of networks. However, the previously researchers of topology control in PDTNs are all not considering the contact opportunity which is an important characteristic in PDTNs.

In this paper, we use the contact opportunity of the nodes of PDTNs in a cycle time to calculate the contact ability, and then construct an undirected weighted contact graph model. The contact opportunity is an important characteristic of PDTNs, and it is very important for transmit data. A contact ability based topology control mechanism is proposed to satisfy the performance requirement while maximizing the contact ability. Our major contributions are summarized as follows:

1. The contact ability model is constructed.
2. The PDTNs for topology control is modeled as an undirected weighted contact graph. The topology control problem is defined as finding the minimum spanning tree (MST) of such a graph. Two algorithms are proposed to find the minimum spanning tree.
3. The constructed MST can maximize the contact ability of transmitting data with the certain energy consumption.

The rest of this paper is organized as follows. In Section “Related works”, we summarize related works in topology control in ad hoc networks and PDTNs. In Section “Node contact opportunity analysis”, contact ability based topology control problem and model are defined. Section “Contact ability based topology control algorithm” describes the details of the heuristic algorithms proposed in this paper. Section “Performance evaluation and analysis” presents the simulation results of the proposed algorithms. Finally, Section “Conclusion” concludes the paper and points out future research directions.

## Related works

### Contact graph based routing in DTNs

Many researches are focus on contact graph based routing in DTNs. Seguí et al.^20^ proposed an Enhancing Contact Graph routing of delay-tolerant spatial networks, which focuses on the analysis of contact graph routing (CGR) based on spatial networks. CGR uses the predictability of contacts to make routing decisions. In their simulations, scenes of similar missions to Mars and the Moon were used to collect statistics on routing protocol performance in terms of latency and buffer usage. They improved the basic cost function of CGR to avoid routing loops and suggested using Dijkstra's shortest path algorithm for path selection. Bezirgiannidis et al.^21^ proposed CGR with the earliest transmission opportunity (CGR-eto) and overbooking management. While evaluating the enhancement effect of the original CGR, they also introduced an experimental version of CGR-eto, which uses the information of local routing data to calculate the queuing delay of all hops that reach the destination through the path, not just the first jump. Caini and Firrincieli^22^ studied the applicability of contact graph routing (CGR) in LEO satellite DTN communication, focusing on two practical application scenarios of earth observation and data mule. The results obtained by running ION, the DTN Bundle protocol and the Linux test bed realized by CGR developed by NASA highlight the advantages of CGR applied to low-orbit satellite communications. Araniti et al.^23^ investigate the Contact Graph Routing in DTN Space Networks, especially the applicability and performance of the DTN protocol stack, and evaluate the experimental experience of the CGR presenting results. Fraire and Finochietto^24^ proposed the challenge of the connection plan of the anti-interruption satellite network. They discussed the increasing complexity of considering routing and traffic information to enrich the planning process, and therefore the need to implement a contact plan calculation element to support spatial DTN operations.

All these contact graph based routing are also focusing on delivering data from source to destination. They do not consider the topology control.

### Topology control for PDTNs

There are few studies on topology control under PDTNs, as far as we know, Huang et al.^11–14^, Chen et al.^15,16^, Yan et al.^17,18^ and Zhang et al.^19^ studied the topology control of PDTNs. Huang et al. modeled the time-evolving network as a weighted space–time graph, which include temporal and spatial information. They proposed an cost efficient topology design (CETD) for PDTNs, and construct a sparse network that meets two goals, that is, every pair of nodes can be connected and the total energy consumption is the smallest. Moreover, the unreliable links is considered in literature^13^. The effectiveness of above proposed topology control methods are all verified by simulation. Yan et al.^17,18^ also modeled the PDTNs as a directed space–time graph that includes spatial and temporal information. They proposed a topology control strategy by using a kth shortest paths algorithm. Zhang et al.^19^ investigated the topology control problem in spacecraft networks where the time-varying topology can be predicted, and they modeled the spacecraft networks as a directed space–time graph, which also includes both temporal and spatial topology information too. They proposed five heuristic algorithms, which can significantly maintain low topology cost efficiency ratio while achieving high reliable connectivity. Although above studies are all construct a sparse network to ensure that each pair of nodes can communicate, but they only consider the spatial and temporal information. The contact opportunity that is an important characteristic in PDTNs is not considered.

To our best knowledge, there are no previous results on topology control based on contact opportunity of PDTNs. This paper is the first attempt to study topology control for PDTNs based on contact ability through constructing MST. We believe that topology can be controlled more wisely and efficiently if the network evolution over time is known.

## Node contact opportunity analysis

In DTNs, the data transmission is achieved through opportunistic contact caused by node motion. Thus, how to define the node contact ability is crucial. In this paper, a network model based on contact graph is established, and the process of node contact with each other under different conditions is analyzed. The contact process of node in single hop and multi-hop is described, and the calculation method of contact probability of each pair of nodes is described. Moreover, the node contact ability measurement is defined.

### Network model

The contact of the node is described by the network contact diagram G (V, E). In the contact graph, V is the set of the nodes, E is the set of the edges. The edge eij represent node i and j can contact. Similar to the literature^25,26^, it is assumed that the contact interval between each pair of nodes follows the exponential distribution of the parameter λij, so the contact process between nodes i and j can be described as the Poisson process with contact rate λij, λij can be calculated by real-time time-average. Although the literature^25^ shows that the node contact time interval is composed of the power-law distribution and the exponential distribution, there is no agreement on the distribution of the contact time of the pair of nodes. The exponential model used is validated in the actual DTN environment given in^27–29^.

**Figure 1:**
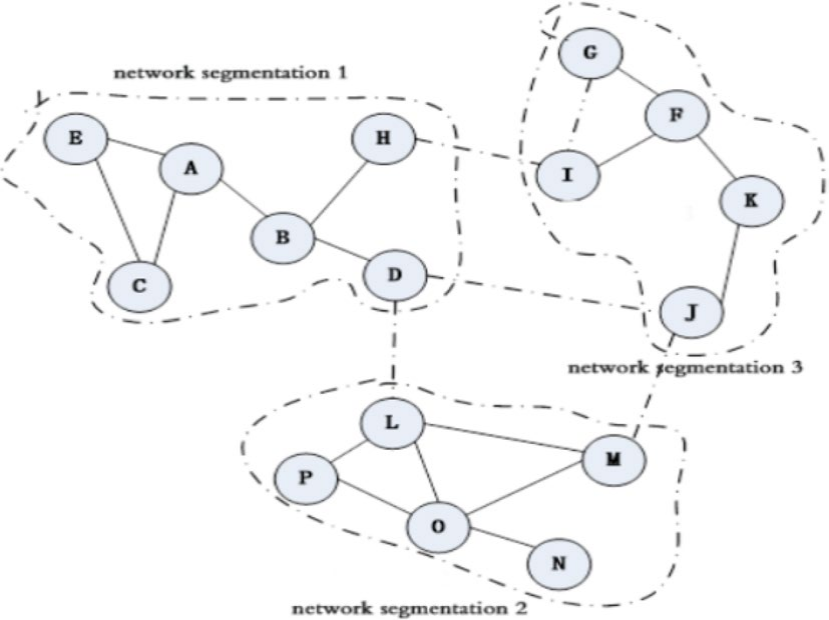
DTN network partitions.

Figure 1 shows an example of a typical DTN network. In DTN networks, due to the randomness of nodes, the links between nodes in a large range are unreliable and intermittent. Due to the nodes movement, the network will be split into many smaller network partitions.

Figure 1 shows the three network partitions 1, 2 and 3, which is circled with virtual line. Within the network partition, the circle with the letter number represents the mobile node, and the solid line of the connected node represents the contact process between the nodes. The dotted line with the connected node represents the node contact process after a period of time. For example, the nodes L, M, N, O, and P belong to the network partition 2, the nodes L and M are contact with λLM contact rate. After a while, node M will leave the network partition 2 and enter the network partition 3, and contact with J by λMJ contact rate.

### Network information

In order to effectively transmit data, it is very important to obtain the network information when the nodes contact. The information include (1) the contact interval between nodes, (2) the node set in contact with the node, (3) the duration of contact between nodes. Due to the instability of the connection in DTNs, it is impossible to share these network information in a global scope, so that the optimal data transmission path cannot be given in the whole network. Thus, exploiting node's local network information to choose the appropriate path to effectively transmit data.

1. Single-hop network information maintenance

**Figure 2:**
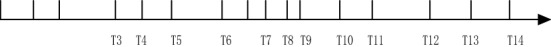
Node contact time.

In the network, each node is identified by an unique ID. When node i contact with node j, the node i will record the node number (here is j), the contact time, and the contact duration, and then put it as a record into their own contact information table. Thus, after a period of time, the contact information table of the node i records its contact history information, and the contact history shown in Fig. 2 can be expressed as the contact table shown in Table 1.

**Table 1:** Node contact information table.

Node number	The contact time	Contact duration
N1	T1	L1
N2	T2	L2
N3	T3	L3
N1	T4	L4
N4	T5	L5
N5	T6	L6
N3	T7	L7
N3	T8	L8

(2) Multi-hop network information maintenance

From the perspective of single-hop to study the contact of the node cannot fully tap the node's mobility and data transmission capacity. For example, node i only contact nodes A and B in a period of time, but node A can contact nodes C, D, and E in the future. When node j needs to send data to E, in the case of single-hop contact, it will not forward data to node i after contact node i, instead continue to wait for the chance of contact with E. Thus, the opportunity to forward data to i via A to E is lost, and reducing the possibility of successful data forwarding, increased access delay. For this reason, it is necessary to consider the contact ability of the node in the multi-hop range.

In the process of maintaining the single-hop network information, the node maintains the multi-hop network information by exchanging the additional network information during the contact process. This information mainly includes the estimated value of the contact rate between the nodes that contact with it and the other nodes. Since the contact condition of the node in DTNs is constantly changing, this information is local, which is called the contact rate estimate (described by Section “Node contact probability(1)”). In this way, each node will maintain two tables, the node contact table and the contact rate estimate table. For a contact rate estimate, all records associated with that node in the table should be updated when the maintenance node contact to the nodes in the table again. That is because the records in the contact rate estimates have expired and need to be updated with the new contact rate estimates to estimate the network contact as accurately as possible.

The following tables are an example of multi-hop network information maintained. Table 2 shows the current node i contact rate estimation table, Table 3 is the contact rate estimation table of node j.

**Table 2:** Contact rate estimation table of node i.

The source node	Target node	Contact rate	Update time
N1	N2	λ1	5
N1	N3	λ2	9
N2	N4	λ3	13
N2	N3	λ4	20
N4	N7	λ5	30
N7	N9	λ6	54

**Table 3:** Contact rate estimation table of node j.

The source node	Target node	Contact rate	Update time
N1	N3	λ7	5
N4	N6	λ8	7
N2	N3	λ9	13
N9	N2	λ10	20
j	N2	λ11	30
j	N11	λ12	54

Node i contact with node j at time 70. First, they add a new contact record between i and j on their respective contact information tables and recalculate the contact rate λij between i and j using the method described in Section “Node contact probability(1)” and update the λij to the contact rate estimate table, the update time is set to 70. Then, node i and j updates the public records in their contact rate estimates and adds new records. The updated results are shown in Tables 4 and 5.

**Table 4:** The updated contact rate estimation table of node i.

The source node	Target node	Contact rate	Update time
N1	N2	λ1	5
N1	N3	λ2	9
N2	N3	λ3	13
N2	N4	λ9	25
N4	N7	λ5	30
N7	N9	λ6	54
N4	N6	λ8	7
N9	N2	λ10	30
j	N2	λ11	46
j	N11	λ12	60
i	j	λij	70

**Table 5:** The updated contact rate estimation table of node j.

The source node	Target node	Contact rate	Update time
N1	N3	λ2	9
N4	N6	λ8	7
N2	N4	λ9	25
N9	N2	λ10	30
j	N2	λ11	46
J	N11	λ12	60
N1	N2	λ1	5
N2	N4	λ4	13
N4	N7	λ5	30
N7	N9	λ6	54
i	j	λij	70

Tables 2 and 3 are only part of i and j's respective contact rate estimates, after i and j contact, they will update the common record (here the second and fourth rows of Table 2, and update the contact rate of N1, N3 in Table 3 to λ2 according to the update time of these common records, the contact rate of N2 and N4 in Table 2 is updated to λ9 and the update time is modified. Next, insert the remaining records in Table 3 into Table 2, and insert the remaining records in Table 2 into Table 3. Finally, the contact rates between i and j are inserted in Tables 2 and 3 and the update times are set to their contact time 70.

**Figure 3:**
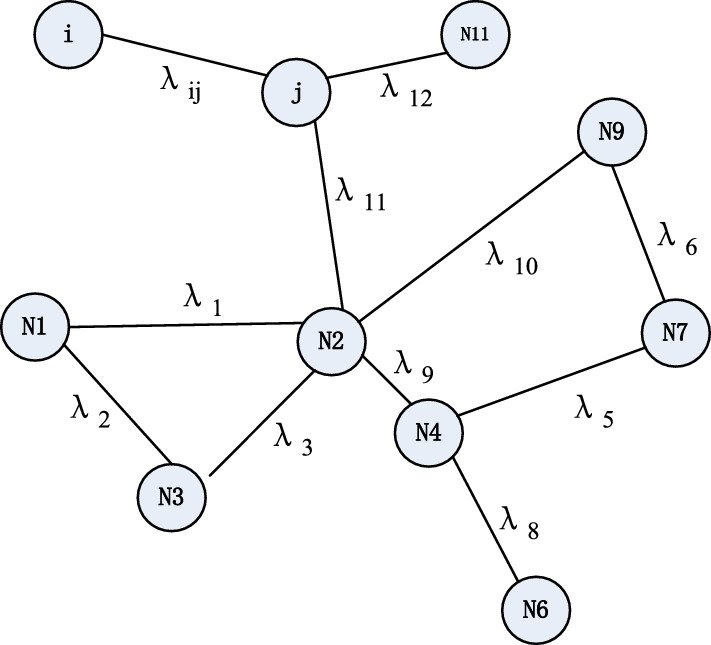
The network contact graph Gi of node i.

After exchanging network information between node i and j, the local contact map Gi is established according to their own maintenance of the network information. Figure 3 shows a schematic representation of Gi.

### Node contact probability

In this paper, the contact ability of nodes is measured through the probability ways. As can be seen from the network model in Section “Network model”, it is assumed that the contact of each pair of nodes is subject to the Poisson process. The node i calculates the contact rate λij with the node j based on the network information maintained by itself. In this section, the contact rate between nodes is considered from the perspective of single-hop and multi-hop.

1. Calculating inter-node contact rate

As described in Section “Network information(1)”, each node maintains a contact information table that records the contact history information of the node, including the contact node ID, contact duration, and so on. In this paper, it is assumed that the contact between nodes obeys the Poisson process and the respective contact rates need to be calculated for each node in the contact information table. For node i, the specific method is as follows:

1. The node numbers in the contact information table are merged into the set S.
2. For each of the set numbers s in the collection S, filter out all the records of the node s in the contact table, and define these records as recordset Rs, which size is Ls.
3. Find the maximum and minimum values of the contact time columns in Rs, denote Tsmax and Tsmin respectively, find the difference Trange of Tsmax and Tsmin, the result of dividing Trange by Ls-1 takes the reciprocal to obtain the contact rate λ is of node i and s.
  1 $$\lambda_{is} = \frac{{L_{s} - 1}}{{T_{s\max } - T_{s\min } }}$$
4. Repeat the process step 2) until all the nodes in S executed in step 2) operation.

(2) Single-hop node contact probability

After knowing the contact rate λij between the nodes i and j, the contact probability between single-hop nodes can be calculated. According to the foregoing assumptions, the contact interval between i and j obeys the exponential distribution of the parameter λij. Assuming that the current time is t, the lifetime of the data copy d is T (from 0 onwards). From the probability density function of the exponential distribution, we can see that the probability of i and j before T is the following formula.

2 $$P(X \le T_{d} ) = 1 - e^{{ - \lambda_{ij} (T_{d} - t)}}$$

where X is the next contact time of i and j.

(3) Multi-hop node contact probability

#### Definition 1

The r-hop opportunistic path PAB = (VP, EP) between A and B, which contains the node set Vp = (A, N1, N2, …, Nr-1, B) and the edge set Ep = (e1, e2, …, er) , the weights are (λ1, …, λr) .

At the same time, the path weight is defined as the probability that the data is forwarded from A to B along the PAB within time T.

**Figure 4:**
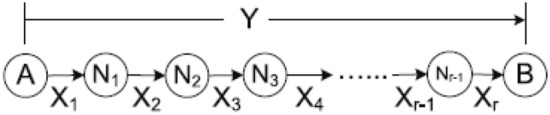
Opportunity path diagram.

As shown in Fig. 4, the contact interval Xk of the nodes Nk and Nk + 1 obeys the exponential distribution of the parameter λk. Whereby the total time required for forwarding the data from A to B is Y = $\sum\nolimits_{k = 1}^{r} {N_{k} }$ obey the super exponential distribution^30^.

#### Lemma 1

^31^ The probability density function (PDF) for Y is a r-hop path of λ1, λ2, …, λr for the edge.

3 $$p_{Y} (x) = \sum\limits_{k = 1}^{r} {C_{k}^{(r)} p_{Xk} (x)}$$

among them, coefficient $C_{k}^{(r)} = \prod\limits_{s = l,s \ne k}^{r} {\frac{{\lambda_{s} }}{{\lambda_{s} - \lambda_{k} }}}$.

From Definition 1, the path weights can be written as the following formula.

4 $$p_{AB} (T) = \int_{0}^{T} {p_{Y} (x)dx} = \sum\limits_{k = 1}^{r} {C_{k}^{(r)} \cdot (1 - e^{{ - \lambda_{k} T}} )}$$

(4) Measurement of contact probability between nodes

With the above description of single-hop contact probability and multi-hop contact probability, it is possible to further analyze the possible contact patterns between nodes.

**Figure 5:**
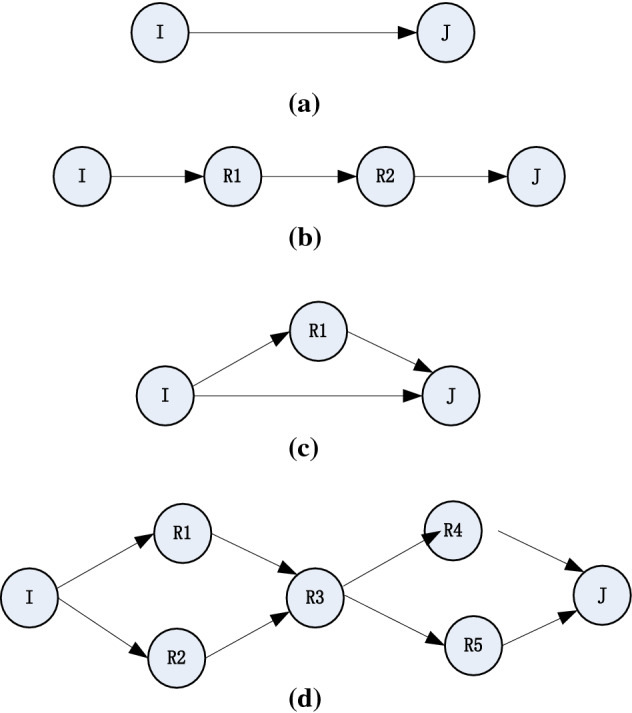
The probability of contact in different situations.

Figure 5 shows the typical ways in which three nodes are exposed. Among them,

1. Figure 5a describes a simple single-hop contact, that is, the node i, j without the help of other nodes in the case of independent contact with each other.
2. Figure 5b describes the case where the nodes i, j are contacted by means of an opportunistic path. In these cases, the contact between i and j requires a series of intermediate nodes, and the contact of these intermediate nodes directly affects i, j's contact probability.
3. Figure 5c describes a case where (a) and (b) are mixed, where there is a direct contact between i and j, with the presence of one or more jumper paths, which makes i, j probability is greater than the probability of direct contact or through the opportunistic path, but the probability calculation in this case becomes complicated. In the initial stage of the network, the information of the network is less, the difficulty of the calculation of the contact probability will be relatively small, but with the continuous accumulation of network information, the complexity of the network will continue to increase, the corresponding calculation will increase the difficulty;
4. The situation in Fig. 5d is more complicated. In this case, there are multi-hop opportunistic paths between nodes i and j and there are common nodes on these paths, which have a very large effect on the whole contact probability. When a public node disappears for some reason in the network, the edges of the network contact graph associated with the node will disappear and the corresponding contact map between i and j will change dramatically. The update of the network information cannot be quickly broadcast in the network, then Fig. 5d in this case the probability of the calculation will be very large deviation.

Through above analysis, we can know that the probability of contact between nodes is different in different situations. In this paper, for the sake of simplicity, this paper does not attempt to calculate the node contact probability in the case of Fig. 5c,d. The Dijkstra algorithm is used to derive the opportunity path with the shortest number of hops, and the basic probability of the path is used as the contact probability between nodes.

### Contact ability calculation

For a given node, its ability to reach other nodes is one of the most important features in the DTN network.

Researchers have proposed a number of ways to tap the node's ability to contact, these methods are centralized, but also distributed. The centralized method assumes that the nodes in the network know the global information of the network. Using this information, the nodes judge the strength of their contact ability in the whole network and pass the data to the nodes with the best or relatively good contact ability. The advantage of this approach is the decision-making accuracy, good performance, the disadvantage is the need for additional means in the whole network broadcast network information. In the distributed method, the node uses the historical contact opportunity to exchange the network information, through the existing network information processing to estimate the network, calculate their own contact ability and in contact with other nodes in contact with other contact ability, thereby deciding whether to forward data.

As described in Sects. Section “Network information(1)” and “Network information(2)”, each node maintains a node contact table and a contact rate estimate. According to the method of Sections “Node contact probability(1)” and “Node contact probability(2)”, the contact information table can be used to calculate the probability of single-hop contact between nodes. According to the description of Section “Node contact probability(4)”, the contact rate estimation table can be used to select the opportunity path between nodes to calculate the multi-hop contact probability. In this paper, we discuss the probability of single-hop and multi-hop contact as the probability of contact between nodes.

#### Definition 2

The node i contact ability measure C_i_ is described by the following formula.

5 $$C_{i} (T_{d} - t) = \sum\limits_{j \in Ni}^{{}} {p_{ij} (T_{d} - t)}$$

where T_d_ is the lifetime of the data copy d from time 0, t is the current time, and Ni is the set of nodes that can be reached. P_ij_ (T_d_-t) is the probability of the contact of i and j in the time T_d_-t from the time t, and the calculation method has been described above.

From the above equation, C_i_(T_d_-t) calculates the expected value of the number of nodes that the node i may be exposed to in future T_d_-t. The expected value reflects the ability of node i to contact the node during the remaining lifetime of replica d.

### Undirected weighted contact graph model

Firstly, according above formula 5, the contact ability of node i can be counted out at a cycle time, where T_d_ is the lifetime of the data replication d from time 0, t is the current time, and Ni is the set of nodes that can be reached. In this paper, we set the current time is 0 and T_d_ is a cycle time of PDTNs. P_ij_ (T_d_-t) is the probability of the contact of i and j in the time T_d_-t from the time t.

And then average the contact ability of each pair of nodes contact with each other in the networks.

**Figure 6:**
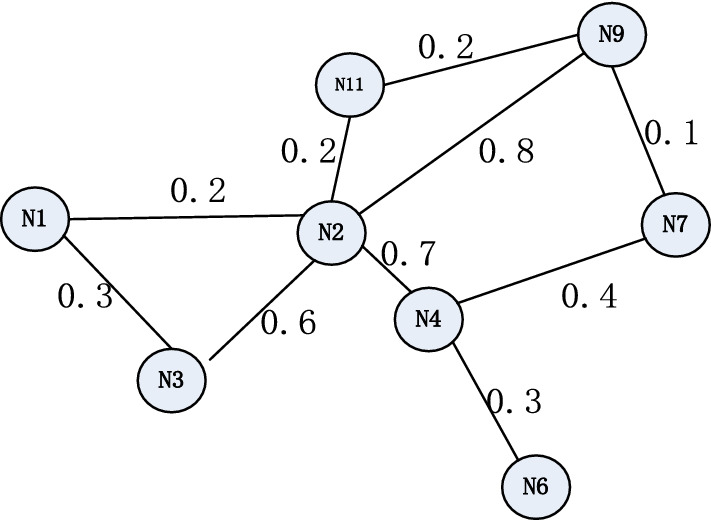
Undirected weighted contact graph.

Finally, according the above calculating contact ability of each pair of nodes, one undirected weighted contact graph is constructed. Such as the following Fig. 6, the vertex represent the nodes of the PDTNs, the edge of the nodes represent the two nodes can be contact, and the weight of the edge is the reciprocal of the average contact ability of the two nodes.

Thus, the total contact ability of the MST is represented by C(C) shown in the following formula:

$${\text{C}}({\text{C}}) = \sum\limits_{K = 1}^{N - 1} {\frac{1}{{W({\text{ViVj}})}}}$$

(ViVj $\in$ G , ViVj is in the minimum spanning tree, *W*(ViVj) is the average contact ability of node i and j, and N is the total number of the vertices of G).

### Energy model

In PDTNs, data transmission requires energy, and energy cost is a very important issue. Minimizing the energy cost is the important goal of topology control, thus an energy consumption model needs to be established. Generally, the free space propagation model Pt(d) ∝ d^2^ is widely used. The Pt is the power of the transmitted signal and d is the Euclidean distance between the transmitter and the receiver. In order to simplify the implementation, according to the distance between each pair of nodes, the energy consumption value of an appropriate edge is given from 1 to 5. We use C(ViVj) to represent the energy cost of communication between node i and node j, and the total energy cost of MST is represented by C(G), as shown in the following formula:

$${\text{C}}({\text{G}}) = \sum\limits_{K = 1}^{N - 1} {C(V{\text{i}}V{\text{j}})}$$

(ViVj $\in$ G , ViVj is in the minimum spanning tree and N is the total number of the vertices of G).

### Problem statement

The aim of topology is to maintain network connectivity with the minimum energy cost. In this paper we mainly focus on the nodes can transmission each other efficiently in the maximum contact ability in PDTNs. Topology control problem can be defined as a tree construction problem, which is finding the minimum spanning tree of undirected weighted contact graph model, such that 1)include all nodes of the network; 2)the contact ability of MST is maximized; and 3) the total energy cost of the MST is minimized. Given a Undirected graph G(V, E, e) , the problem is to find a MST of G(V,E, e) that satisfies,

$$\begin{aligned} & {\text{maxmize:}}\,\,{\text{C}}({\text{C}}) \\ & {\text{subject}}\,{\text{to:}}\,\,{\text{C}}({\text{G}})\,{\text{is}}\,{\text{minimize}} \\ \end{aligned}$$

## Contact ability based topology control algorithm

### Variant Kruskal Algorithm base on Undirected Weighted Contact Graph (VKAUWCG)

The basic idea of the first algorithm is finding the biggest contact ability edges that satisfy the energy cost is minimize to ensure all nodes of the networks can transmission each other efficiently.

The VKAUWCG contains the following steps:

Step 1: All the edges are sorted in the ascending order according to the reciprocal of the average contact ability of each pair of nodes defined in the undirected weighted contact graph. And every vertex of the entire network is initiated as a connected component.

Step 2: Choosing the undirected edge with the smallest weighted edge, if the smallest weighted edge is more than one, then select the smallest energy cost edge, and merge the vertexes of this edge as one connected component.

Step 3: Choosing the undirected edge with the second smallest weighted edge. If the second smallest weighted edge is more than one, then select the smallest energy cost edge. If the vertexes of this edge satisfy the certain conditions, they will be merged into the previous connected component. These conditions are:

(1) The vertexes are in different connected component.

(2) The vertexes haven’t connected with the edges.

Step 4: If the above conditions are not satisfied, the undirected edge with the ith(i = 3,… ) smallest weighted edge is selected and Step 3 is performed repeatedly until all vertexes have been reached by the selected edges. If one connected component contains all these vertexes, this connected component is the Minimum Spanning Tree (MST). Otherwise, the network is not complete connected.

Step 5: The contact ability and the ratio of total cost are calculated. And this algorithm ends with the established MST.

The pseudo code of VKAUWCG algorithm is as follows, in which CC represents the connected component, Ne represents the number of the edge, V(E_i_) represent the two vertexes of edge E_i_, n represents the number of the vertex:
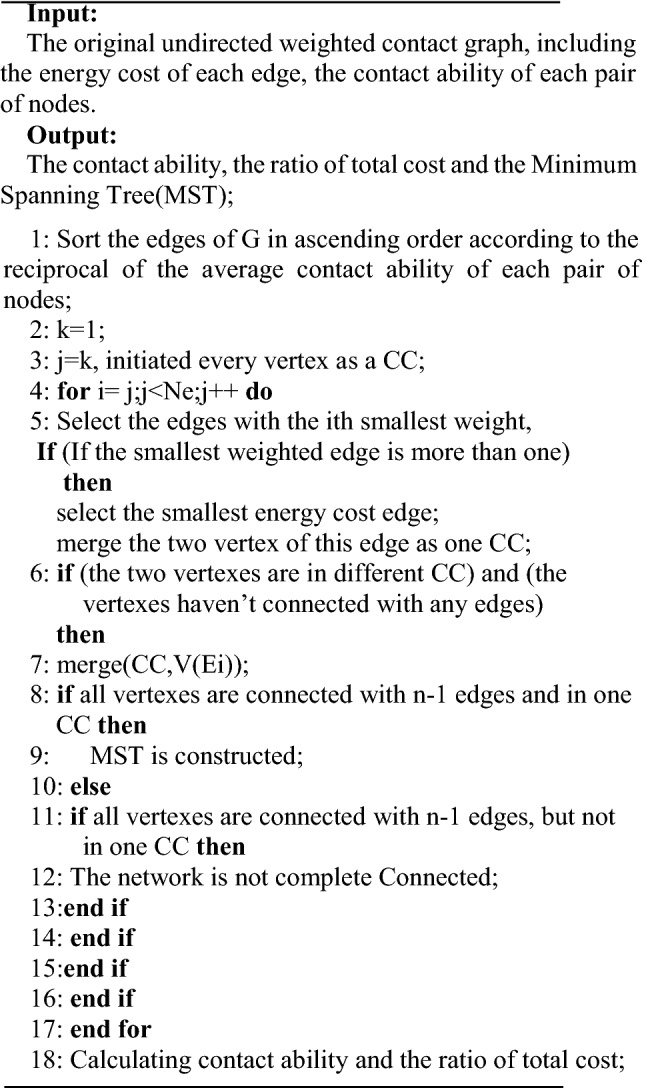


The time complexity of line 1 is O(n^2^), the time complexity of line 4–16 is O(n × logN_e_). So the time complexity of the algorithm is O(n^2^ + n × logN_e_).

#### Theorem 1

The time complexity of the above algorithm is O(n^2^ + n × logN_e_), in which n represents the number of the nodes in the network, N_e_ represents the number of the edges of the network.

### Variant Prim algorithm base on Undirected Weighted Contact Graph (VPAUWCG)

The basic idea of the second algorithm is to find the biggest contact ability edges that satisfy the energy cost is minimize to ensure all nodes of the networks can transmission each other efficiently by adding nodes. The VPAUWCG contains the following steps:

Step 1: The connected component (CC) is initiated as empty. One vertex is randomly chosen and added to the CC.

Step 2: Choosing the undirected edge with the smallest reciprocal of the average contact ability between nodes connected to the CC’s vertex. And to ensure that no closed cycle will form after joining this edge.

Step 3: If the smallest weighted edge is more than one, then select the smallest energy cost edge. The connected vertex of the selected edge is added to the CC if it is not in the CC.

Step 4: If CC still does not contain all the vertexes, goto Step 2 until all vertexes are in the CC.A MST is constructed. Otherwise, the network is not complete connected. Goto Step 1.

Step 5: The contact ability and the ratio of total cost are calculated. And this algorithm ends with the established MST.

The pseudo code of VPAUWCG algorithm is as follows:
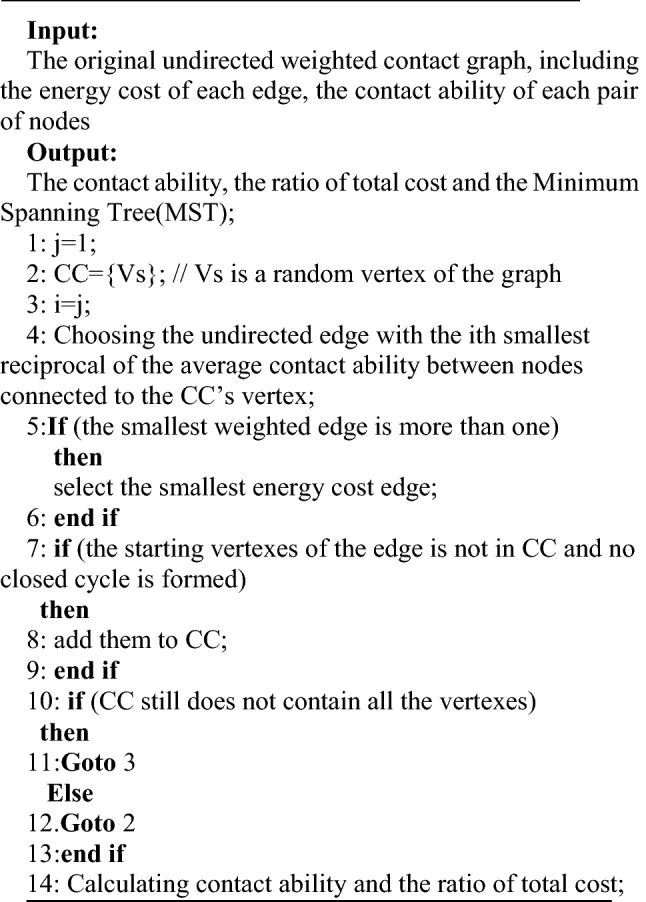


The time complexity of line 4 is O(N_e_ × logn), and for line 5–13 is O(n × N_e_ × logn). So the time complexity of the algorithm is O(n × N_e_ × logn).

#### Theorem 2

The time complexity of the above algorithm is O(n × N_e_ × logn), in which n represents the number of the node in the network, and N_e_ represents the number of the edges of the network.

## Performance evaluation and analysis

In this section, we will evaluate and analyze our proposed algorithms, namely, Variant Kruskal Algorithm base on Undirected Weighted Contact Graph (VKAUWCG) and Variant Prim algorithm base on Undirected Weighted Contact Graph (VPAUWCG) by comparing their performance in terms of the energy cost and contact ability. We compared our algorithms with GrdLCP^14^ and our previous MSTRAG^15^ and VPASPG^16^. We implement all these algorithms in a simulated environment. The underlying PDTNs are randomly generated from a random graph model or directly extracted from Cambridge Haggle tracing data^32^.

In simulation, we mainly consider the following performance metrics and parameters:

1. The ratio of total cost: the performance metric is mainly used to evaluate whether the algorithm is good or bad by the energy efficiency, the smaller the total energy cost, the algorithm is more optimal.
  The ratio of total cost = total energy cost value/the whole network energy value.
2. Link density (ρ): It refers to the sparse/dense degree of the network topology.
  Link density = the number of links of current topology/ the number of links of the corresponding complete graph of the current topology.
3. Contact ability: It refers to the contact ability if each pair of nodes. In this paper the contact ability can be obtained from formula 5.

### Simulations on random networks

We use the undirected weighted contact graph to represent the predictable DTNs. It has 10 nodes and the distribution of nodes is different for each run. The cost of each edge is randomly chosen from 1 to 5, and the reciprocal of the contact ability of each edge is randomly chosen from 0 to 1. For all the simulations, we repeat the experiment for 100,000 times and report the average values of the metric.

**Figure 7:**
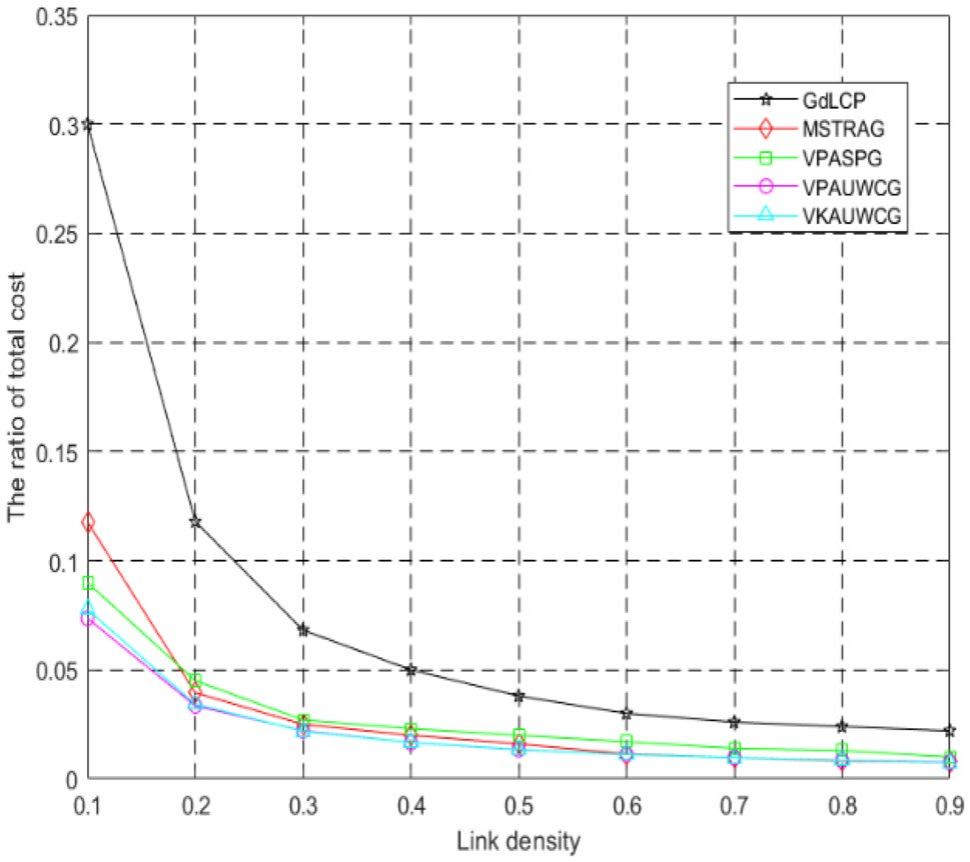
Ratio of total cost of different link density ρ.

Figure 7 depict the impact of link density ρ to the ratio of total cost. As can be seen from the figures, our two algorithms proposed in this paper are always better than the other algorithms, especially when the link density is smaller. However, with the link density increasing VPASPG and MSTRAG proposed in our previous research is very similar to our two algorithms proposed in this paper. That is because the methods proposed in our paper are constructing a tree for topology control. From Fig. 7 we can also see the ratio of total cost decreases with ρ increasing. Even when ρ is 0.1, the ratio of total cost is in the range of 0.063 ~ 0.07, which indicates that even in the sparse network especially in the challenge environment our algorithms could save more energy.

**Figure 8:**
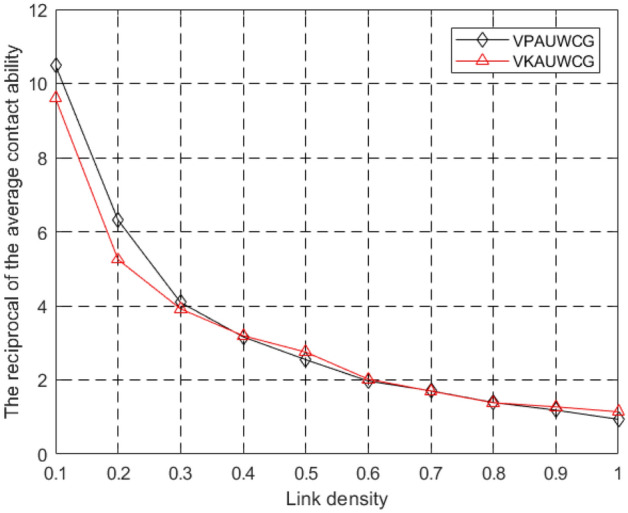
The reciprocal of the average contact ability of different link density ρ.

**Figure 9:**
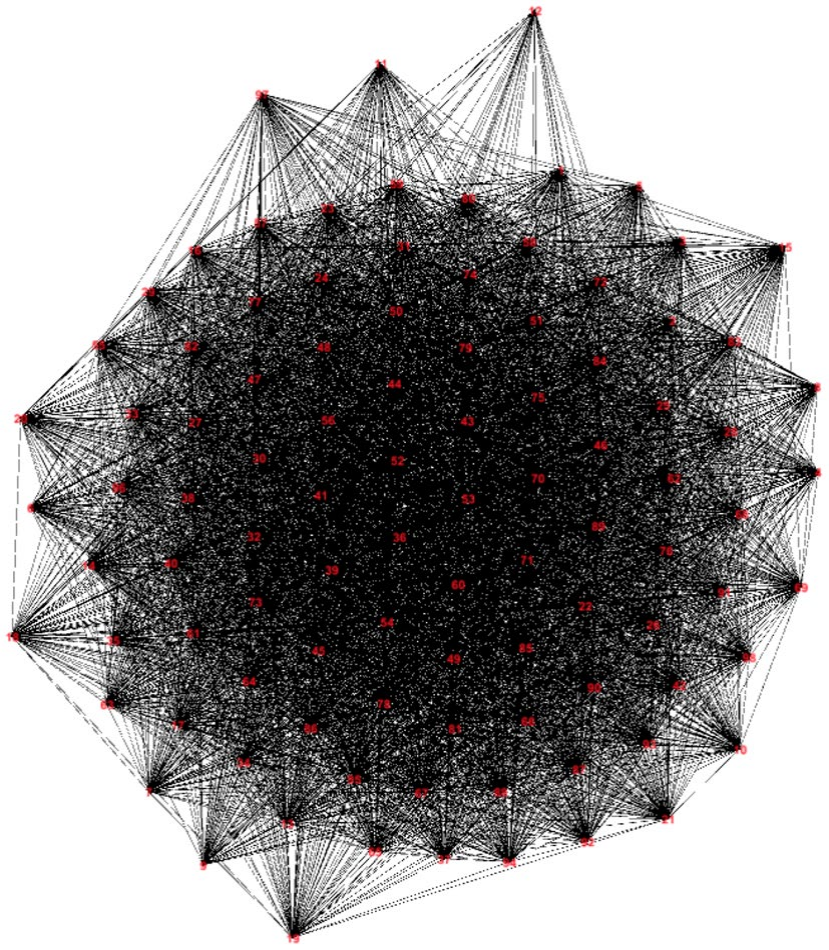
The constructed original graph of the Tracing Data using our model.

Figure 8 depict the impact of link density ρ to the reciprocal of the average contact ability. As can be seen from the figures. The tendency of the reciprocal of the average contact ability of the two algorithms proposed in this paper decreases with ρ increasing. which indicated that the contact ability of the network is increasing with ρ increasing. That is because with the link density increasing, the density of the networks is very high, thus the contact ability is very big. From Fig. 8 we can also see the reciprocal of the average contact ability is very low when ρ is small. Even when ρ is 0.1 , the reciprocal of the average contact ability is in the range of 9–11, which indicates that even in the sparse network especially in the challenge environment our algorithms could ensure the data can be transmitted to the other nodes efficiently. That’s because in our simulation the network has 10 nodes. Figure 9 also depict VKAUWCG is better than VPAUWCG when ρ is smaller than 0.3. Thus, if the required contact ability is large, we can use the VKAUWCG, otherwise if the required energy cost is small, VPAUWCG will be used.

### Simulations on tracing data

In this paper, we use the data sets from Cambridge Haggle data^31^. In this data set, connections among 78 mobile iMote Bluetooth nodes carried by researchers and additional 20 stationary nodes are recorded over four days during IEEE Infocom 2006. In our simulation, we only consider the 78 mobile nodes, if there is a contact between each pair of two nodes, an edge is added to the graph. The weight of the edge is represented by the reciprocal of the sum of the number of contacts of the two nodes. The following Fig. 9 is the constructed original graph of the Tracing Data using our model.

From the original graph of the network, we can see the graph is very dense. If we don’t topology control for it, the energy cost will be very large, and the signal interference will be strong. Thus topology control is very necessary.

**Figure 10:**
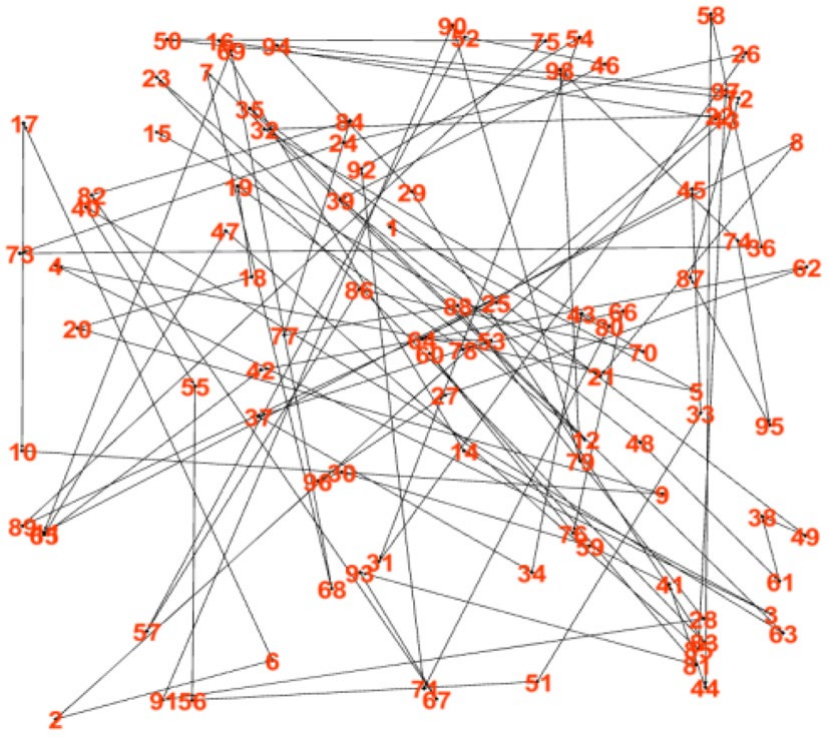
The constructed graph using the contact ability based topology control methods.

Figure 10 is the constructed graph using the contact ability based topology control methods proposed in this paper. From the results, we can see the method proposed in this paper is very efficient in terms of energy cost and contact ability. The graph become very sparse, the contact ability is very high, and the energy cost is very small. Due to the links of the network is very less, thus the signal interference is also very small.

## Conclusion

In this paper, we model PDTNs as an undirected weighted contact graph which includes spatial and contact ability information and defined the topology control problem as finding a Minimum Spanning Tree(MST) from the graph model to maximize the contact ability. We propose two heuristic topology control algorithm, VKAUWCG and VPAUWCG to solve this problem.

The next step is to consider the following problems: (1) Solving the topology control problem for unpredictable DTNs; (2) Devising a method that can get contact information accurately; (3) Dealing with topology control of PDTNs when some node may fail in the movement; (4) Balancing energy cost of the network.
